# Oxycodone attenuates endotoxin-induced acute lung injury by regulating mitophagy via the HO-1 pathway

**DOI:** 10.1016/j.clinsp.2025.100753

**Published:** 2025-08-23

**Authors:** Cuicui Liu, Yanting Wang, Shaona Li, Jia Shi, Pei Wang, Yang Ma, Xiangkun Li, Jianbo Yu

**Affiliations:** a Tianjin Nankai Hospital, Tianjin Medical University, Tianjin, China; b The Affiliated Hospital of Qingdao University, Qingdao, Shandong, China; c Institute of Integrative Medicine for Acute Abdominal Diseases, Tianjin, China; d Tianjin Key Laboratory of Acute Abdomen Disease Associated Organ Injury and ITCWM Repair, Tianjin, China; e The Affiliated Hospital of Chengde Medical University, Chengde, Hebei, China

**Keywords:** Oxycodone, Endotoxin, Acute lung injury, Mitophagy, Heme oxygenase-1 (HO-1)

## Abstract

**Background:**

Endotoxin-induced Acute Lung Injury (ALI) is a severe clinical syndrome with limited treatment. Oxycodone can alleviate the endotoxin-induced ALI, but the exact mechanism remains unclear. The previous study showed that Heme Oxygenase-1 (HO-1) plays a protective role against endotoxin-induced ALI by regulating mitophagy. Thus, the authors aimed to elucidate whether oxycodone attenuates lung injury by regulating mitophagy via the HO-1 pathway.

**Methods:**

Lipopolysaccharide (LPS) ‒ stimulated mice and Mouse Lung Epithelial (MLE12) cells were used to create the model of ALI. After pre-treatment with Oxycodone for the LPS-induced model *in vivo* and *in vitro*, markers of cell and tissue damage, oxidative stress, inflammation, and mitophagy were detected. HO-1 knockout mice and HO-1 siRNA *in vitro* were used to further clarify the role of Oxycodone.

**Results:**

Pre-treatment with oxycodone could alleviate lung pathological damage, reduce oxidative stress and inflammatory cytokines, increase the expression of HO-1 while down-regulate the mitophagy-related proteins (PINK1, Parkin, LC3 II/I). Furthermore, treatment with oxycodone in HO-1-knockout mice or HO-1 siRNA-transfected MLE12 cells revealed the protective role of the HO-1 pathway on oxycodone-mediated mitophagy in LPS-induced ALI.

**Conclusion:**

HO-1 deficiency partially counteracts the beneficial effects of oxycodone on pulmonary protection and inhibition of mitophagy both *in vivo* and *in vitro*. Oxycodone pretreatment attenuated LPS-induced systemic inflammation and subsequent lung injury by regulating mitophagy via the HO-1 pathway.

## Highlights

• Oxycodone alleviated the LPS-driven lung pathological injury both *in vivo* and *in vitro*.
• Oxycodone pretreatment attenuated LPS-induced systemic inflammation and subsequent lung injury.
• Oxycodone increases the expression of HO-1 while downregulating the mitophagy-related proteins.
• HO-1 deficiency weakens the effect of oxycodone on the expression of mitophagy-related proteins.
• Oxycodone attenuates LPS-induced cell injury by regulating mitophagy via the HO-1 pathway.

## Introduction

Acute Lung Injury (ALI) or its more serious form, Acute Respiratory Distress Syndrome (ARDS), is one of the most serious clinical syndromes of lung injury and the leading cause of acute respiratory symptoms.^1^^,^^2^ Despite years of research on ALI, its incidence rate remains high in the intensive care units with a mortality rate of 35%–46%, but there is a lack of effective treatment.^3^ ALI can be caused by several conditions, such as sepsis, Lipopolysaccharide (LPS) exposure, ischemia-reperfusion injury, or trauma, and/or other factors, and is characterized by diffuse alveolar damage and the formation of pulmonary edema.^4^^,^^5^ At present, although the pathogenesis of ALI is not fully understood, the inflammation is considered to be an important pathogenic factor of ALI.^6^

Mitochondria are the energy center of eukaryotic cells, and it is an essential organelle for metabolism, energy conversion, and signal transmission in cells. They are essential for maintaining the normal physiological function of cells and the ability of cells to respond to stress in the environment. The dynamic disorder and dysfunction of the mitochondria is proven to be one of the important pathogenic factors in the process of oxidative stress in ALI.^7^^,^^8^ In addition, mitophagy has also been confirmed to be involved in the pathological process of ALI. The functional status of mitochondria depends on the dynamic balance between mitochondrial generation, fusion and fission, as well as the autophagy-related degradation of damaged mitochondria. Autophagy is a highly conserved process of degradation. Intracellular mitophagy plays an important role in maintaining normal mitochondrial function by degrading and removing the damaged mitochondria.^9^^,^^10^ However, the excessive mitophagy may promote mitochondrial dysfunction, leading to cell injury and death.^11^ Several pathways were reported to be involved in the process of mitophagy, among which, the PTEN-Induced Kinase-1 (PINK1) / Parkin-mediated pathway is known to be a classical pathway.^12^^,^^13^

Heme Oxygenase-1 (HO-1) is widely expressed in different tissues and cells of the body, which can degrade heme and then produce cytoprotective byproducts such as ferritin, biliverdin, and carbon monoxide.^14^^,^^15^ As an endogenous antioxidant protein, HO-1 has been shown to have anti-inflammatory and antioxidant properties in sepsis-related ALI.^7^^,^^16^^,^^17^ Moreover, the previous study showed that it can also play an endogenous protective role in endotoxin-induced ALI by regulating mitophagy-related PINK1/Parkin mediators.^18^

Some previous studies have shown that opioids can reduce systemic inflammation and improve ALI. Morphine pretreatment can improve LPS-induced lung injury, inhibit lethal endotoxic shock, and improve the survival rate of mice.^19^ Sufentanil can reduce inflammation and oxidative stress in sepsis-induced ALI by down-regulating the KNG1 expression.^20^ These studies suggested that ALI can be improved by inhibiting inflammation and oxidative stress.

Oxycodone, a semi-synthetic opioid extracted from thebaine that is widely used as an analgesic to treat post-operative patients and other acute pain in adults. It is a dual agonist of μ- and κ-opioid receptors that can regulate cytokine levels and inhibit inflammation and oxidative stress. In addition to the research on pain treatment, some current research on oxycodone also focuses on the protection of organs such as the heart, brain and lungs. For example, Xie et al.^21^ reported that oxycodone can inhibit myocardial apoptosis after myocardial ischemia-reperfusion injury in rats via the RhoA/ROCK1 signaling pathway. In addition, oxycodone pre-administration can significantly attenuate hippocampal neuronal apoptosis induced by focal cerebral ischemia-reperfusion in rats.^22^ In terms of lung protection, oxycodone has been shown to improve lung damage induced by LPS and mechanical ventilation by inhibiting inflammation and apoptosis.^23^ However, the mechanism of oxycodone in lung protection remains unclear. A recent study showed that oxycodone can alleviate the damage of human endometrial stromal cells by activating the keap1/Nrf2/HO-1 signaling pathway,^24^ suggesting that there may be a certain correlation between oxycodone and the HO-1 pathway.

Herein, the authors used LPS-stimulated ALI mice and cell models *in vitro* to determine whether oxycodone can ameliorate endotoxin-induced ALI by regulating mitophagy. Furthermore, HO-1-deficient or knockdown mice and HO-1 siRNA-transfected Mouse Lung Epithelial (MLE12) cells were used to determine whether the HO-1 signaling pathway is involved in the regulatory role of oxycodone in endotoxin-induced ALI.

## Materials and methods

### Animals

Two-month-old healthy C57Bl/6J mice weighing 20–25g were provided by the Laboratory Animal Center of Nankai Hospital, Tianjin, China. The characterized HO-1 conditional knockout (^−/−^) mice with C57BL/6J background (HO-1^fl/fl^/CAG-CreERT2) were obtained from Beijing Biocytogen Co., Ltd... Around 3–5 mice were housed per cage at a temperature of 22–25°C and humidity of 50%–65%. They were acclimatized to a 12h light-dark cycle with ad libitum access to food and water.

All animal care and experimental procedures of mice were conducted according to the National Institutes of Health Guidelines with the approval of the Animal Ethical and Welfare Committee of Tianjin Nankai Hospital (nº NKYY-DWLL-2022-049). The animal study followed the ARRIVE guidelines. All experiments were performed in specific pathogen-free animal laboratories.

### Establishment of LPS-induced ALI model

Systemic LPS administration is a well-established model for studying ALI. This model has been frequently established in these previous studies,^7^^,^^16^, ^17^, ^18^ and the authors found that systemic administration of LPS could cause a systemic inflammatory response, thereby inducing a satisfactory ALI model. The detailed steps are expounded as follows. After 12h of fasting without restricting water intake, the mice underwent anesthetic induction with 2% sodium pentobarbital (50 mg/kg) by intraperitoneal injection, which was maintained by inhalation of 1.5%–3% isoflurane. Thus, mice were kept in an anesthetized state throughout the experiment. In the first part of the experiment, the mice were randomly divided into four groups (n = 6/group): control group, LPS group, LPS + OXY group, and OXY group. In the following experiment, the wild-type and knockout mice were randomly divided into three groups (n=6/group): Control group (Control), ALI group (LPS), oxycodone plus LPS group (LPS + OXY), HO-1 Knockout group (HO-1 KO), HO-1 knockout ALI group (HO-1 KO + LPS), and HO-1 knockout plus oxycodone ALI group (HO-1 KO + LPS + OXY). Following the team’s previous approach to establish an endotoxin-induced ALI model,^7^ 15 mg/kg LPS (*E.coli*-L2880, Sigma, USA) diluted in 0.2 mL 0.9% sterile sodium chloride was injected via the caudal vein. Mice in LPS + OXY and HO-1 KO + LPS + OXY groups were pretreated intraperitoneally with 2 mg/kg oxycodone (118298, Mengdi China Pharmaceutical CO., Ltd., China) 30 min before LPS injection.^23^ At 12h after LPS injection, the mice were killed by an overdose of sodium pentobarbital, and blood samples were collected. The lung tissue was harvested and snap-frozen in liquid nitrogen, then stored at -70°C until further examination. The mice grouping and experimental schedule are presented in Table 1.

**Table 1:** Animal groupings and experimental treatments.

**Grouping**	**Treatment**
Effects of OXY on LPS-induced ALI in C57BL/6 J mice (n *=* 6/group)	
Control	Sham operation plus normal saline
LPS	Caudal vein injection of 15 mg/kg LPS diluted in 0.2 mL saline for 12h
LPS + OXY	2 mg/kg OXY was intraperitoneally (i.p.) administered 30 min prior to LPS challenge
OXY	Sham operation plus 2 mg/kg OXY
Effects of the HO-1 pathway on mitophagy during OXY attenuates lung injury in HO-1 knockout (HO-1 KO) and WT mice (n *=* 6/group)	
WT + Control	WT mice + Sham operation plus normal saline
HO-1 KO + Control	HO-1 KO mice + Sham operation plus normal saline
WT + LPS	WT mice + Caudal vein injection of 15 mg/kg LPS diluted in 0.2 mL saline for 12h
HO-1 KO + LPS	HO-1 KO mice + Caudal vein injection of 15 mg/kg LPS diluted in 0.2 mL saline for 12h
WT + LPS + OXY	WT mice + 2 mg/kg OXY was i.p. administered 30 min prior to LPS challenge
HO-1 KO + LPS + OXY	HO-1 KO mice + 2 mg/kg OXY was i.p. administered 30 min prior to LPS challenge

OXY, Oxycodone; LPS, Lipopolysaccharide; ALI, Acute Lung Injure; HO-1, Heme Oxygenase-1; WT, Wild Type; KO, Knockout.

### Cell culture and grouping

MLE12 cells were provided by the American Type Culture Collection (ATCC) and were seeded in 96-well plates at a density of 100 μg/well and then cultured in 10% heat-inactivated fetal bovine serum under a humidified atmosphere of 5% CO_2_ at 37°C. The medium was replaced every 3 days, and cells from passages 2–3 were used for the experiments. The cells were randomly divided into five groups: Control group (Control), ALI group (LPS), oxycodone plus LPS group (LPS + OXY), oxycodone + LPS + HO-1 siRNA group (HO-1 siRNA + LPS + OXY), and oxycodone + LPS + Negative Control (NC) siRNA group (NC siRNA + LPS + OXY). An experimental endotoxemia model was established using an LPS concentration of 10 μg/mL. Oxycodone was given 2h before LPS exposure, and the concentration of oxycodone was determined as 10 μg/mL in the pre-experiment. The gene silencing groups were treated with HO-1 siRNA and NC siRNA transfection for 12h and then challenged with LPS, 30 min after oxycodone was administered. After 24h of co-incubation, both the cells and the cell supernatant were collected for detection (Table 2).

**Table 2:** Cell groupings and experimental treatments.

**Grouping**	**Treatment**
Effects of the HO-1 pathway on OXY-mediated mitophagy during LPS-induced oxidatived injury in HO-1 siRNA-transfected MLE12 cells (n *=* 6 per group)	
Control	Cells are cultured normally in medium
LPS	10 μg/mL LPS in the cultured medium for 24h
LPS + OXY	10 μg/mL OXY pre-incubated 2h prior to 10 μg/mL LPS for 24h
HO-1 siRNA + LPS + OXY	10 μg/mL OXY pre-incubated 2h prior to 10 μg/mL LPS for 24h in HO-1 si RNA-transfected MLE12 cells
NC si RNA + LPS + OXY	10 μg/mL OXY pre-incubated 2h prior to 10 μg/mL LPS for 24h in NC si RNA-transfected MLE12 cells

OXY, Oxycodone; LPS, Lipopolysaccharide; ALI, Acute Lung Injury; HO-1, Heme Oxygenase-1; NC, Negative Control.

### Histological analysis of the lung tissue

The left lung was harvested and stored in 10% neutral buffered formalin, following which the lung tissues were dehydrated, embedded in paraffin, and sectioned to 4 μm-thich slices; the sections were then stained with Hematoxylin and eosin (H&E). The pathological features of every mouse lung were observed under a light microscope (Olympus, Japan). Five slices were taken from each mouse, and five different fields of view were chosen from each slice. Referring to the method adopted by Mikawa et al.,^25^ the degree of lung injury was evaluated in accordance with the pulmonary edema, alveolar congestion, neutrophil infiltration or aggregation, alveolar wall thickening, or hyaline membrane observed. It was then assigned 0–4 points depending on the severity of the lesion: 0- Indicated no lesion or very mild lesions, 1- Indicated mild lesions, 2- Indicated moderate lesions, 3- Indicated severe lesions, and 4- Indicated extremely severe lesions. All the samples were scored by two pathologists who were blinded to the experiment. Finally, the authors added up the scores of each index, and the sum obtained was the total score of lung injury.

### Wet-to-dry weight (W/D) ratio

The right lung tissues were taken, and the surface water was cleaned with filter paper. An electronic scale was used to calculate the weight of the tissue as wet weight. After that, the right lung tissues were dried in an oven at 70°C for over 48h until the weight remained stable, and their weight was determined as dry weight. Wet weight/dry weight was the final W/D ratio.

### TUNEL assay analysis in LPS-induced ALI

Cell apoptosis of the lung tissue was detected using the TdT-mediated dUTP Nick-End Labeling (TUNEL) assay according to the manufacturer’s instructions for In Situ Cell Death Detection Kit (Roche, USA) and DAB detection kit. In brief, after dewaxing and hydration, the lung tissue was immersed in TBS. 0.1% TritonX-100, 0.1% sodium citrate, and 10% goat serum were added to the tissue. After discarding the serum and surrounding tissue fluid, 50 μL Tunel reaction mixture was added to each piece and incubated. Similarly, the nucleus was stained and incubated with 6-Diamidino-2-Phenylindole (DAPI). The slides were sealed and stored in the dark at 4°C and subsequently photographed.

### ELISA quantification of inflammatory factors

The serum and culture supernatant levels of Interleukin-1β (IL-1β), Interleukin-10 (IL-10), and Tumor Necrosis Factor-α (TNF-α) were measured by Enzyme-Linked Immunosorbent Assay (ELISA) kits (IL-1β: SEKM-0002; IL-10: SEKM-0010; TNF-α: SEKM-0034; Beijing Solarbio Science & Technology Co., Ltd., China). Optical densities and standard curves were used to measure the inflammatory factor levels according to the manufacturer’s instructions.

### Immunofluorescence staining analysis

Lung tissues were fixed in 4% paraformaldehyde for 10 min and rinsed twice with PBS. These slices were incubated with primary antibody overnight, followed by fluorescein-coupled secondary antibody for 1h. Rabbit anti-HO-1 antibodies (Proteintech, 66743-1-lg, 1:200 dilution) or anti-LC3 antibodies (Proteintech, 14600-1-AP, 1:200 dilution) were used. Nuclear staining was carried out with DAPI in the dark. The images were captured by a fluorescent microscope (Olympus, Japan).

### Cell viability assay

Cell viability was estimated using the Methylthiazolyldiphenyl-Tetrazolium (MTT) assay. Five groups of MLE12 cells were seeded in 96-well plates at a density of 1×10^4^ cells/mL and cultured overnight at a temperature of 3°C. Then, 10 UL MTT (2.5 mg/mL in 1M PBS) was added to each well, followed by incubation for 3h at 37°C in 5% CO_2_. The Optical Density (OD) absorbance was then measured at 500 nm under a microplate reader (Bio-Rad, Hercules, CA, USA).

### Biochemical analysis

The Superoxide Dismutase (SOD) activity and Malondialdehyde (MDA) contents in the serum and cell supernatant were measured using commercial reagent kits (Nanjing Jiancheng Bioengineering Institute, Nanjing, China) according to the manufacturer’s instructions.

### Mitochondrial reactive oxygen species (ROS) production assay

Mitochondria were extracted from the left lung tissue. Then, 2’,7’-Dichlorofluorescein Diacetate (DCFH-DA) was added to the purified mitochondrial solution at a ratio of 1:1000 as a probe. After incubation in the dark in a CO_2_ incubator at 37°C for 30 min, followed by rinsing in PBS, the fluorescence intensity was measured using a multifunctional enzyme marker. The ratio of 483 nm (excitation) light/535 nm (emission) was considered the final ROS value. The authors also measured the ROS production in lung epithelial cells. Briefly, after 24h of co-incubation with LPS, the lung epithelial cells were incubated with 10 μM DCFH-DA at 37°C for 30 min and then thoroughly washed in PBS. The fluorescence intensity was subsequently measured.

### Mitochondrial DNA content measurement

DNA from lung tissue was extracted using a kit (Aidelab Biotechnologies Co., Ltd, Beijing, China), while DNA from cell samples was extracted using a DNA Micro Kit (Qiagen) in accordance with the manufacturer’s instructions. The copy number of mitochondrial DNA (mtDNA) was measured by real-time PCR according to Amaral A, et al.^26^ The mtDNA was amplified with DN1 encoded by mitochondrial DNA, while the internal reference gene was amplified with β-actin. The reaction mixture comprised 10 μL AceQ Universal SYBR qPCR Master Mix, 0.4 μL forward primer and 0.4 μL reverse primer, 4.2 μL ddH_2_O, and 5 μL DNA in a total volume of 20 μL. The reaction conditions were as follows: pre-denaturation at 95°C for 30s followed by 40 cycles at 95°C for 5 min, 95°C for 10s, and 60°C for 30s. The mtDNA/β-actin DNA ratio of the Control group was defined as 1, and the mtDNA/β-actin DNA ratio of each treatment group was calculated to analyze the content of mtDNA relative copy number.

### Western blot analysis

The expression of proteins in lung tissue and MLE12 cells was measured by western blotting. Lung tissue and cells were lysed in Radio-Immunoprecipitation Assay (RIPA) lysis buffer containing a protease inhibitor. Protein concentration was determined using a Bicinchoninic Acid (BCA) assay kit (Sigma, USA). Equal amounts of soluble protein were separated on 10%–15% SDS-PAGE and then transferred to a polyvinylidene difluoride membrane (Bio-Rad, USA). The membrane was blocked with 5% skimmed milk in Tris-Buffered Saline and Tween 20 (TBST) at 37°C for 2h. Then, the samples were incubated overnight at 4°C with primary antibodies against PINK1 (1:1000, ab300623); Parkin (1:2000, ab77924); LC3 (1:2000, ab19289); HO-1 (1:1000, ab305290); and β-actin (1:2000, ab179467). Next, the blots were washed thrice in TBST, followed by incubation with appropriate horseradish peroxidase-conjugated anti-rabbit immunoglobulin G (1:3000) at room temperature for 1h. The protein densities were normalized to β-actin, and the blots were visualized using an enhanced chemiluminescence detection system (Bio-Rad).

### Statistical analysis

All experiments were performed at least in triplicate. Values were analyzed using GraphPad Prism 9.2.0 (GraphPad Software, La Jolla, CA) and expressed as mean (SEM) or medians (range). The Mann-Whitney *U* test was used to analyze the lung injury scores. For comparisons among multiple groups, the statistical significance was determined by one-way analysis of variance (ANOVA), or two-way ANOVA followed by Bonferroni’s post-hoc correction test. A p < 0.05 was considered to indicate statistically significant differences.

## Results

### Oxycodone alleviated the LPS-driven lung pathological injury, inflammation and oxidative stress in mice

**Fig. 1:**
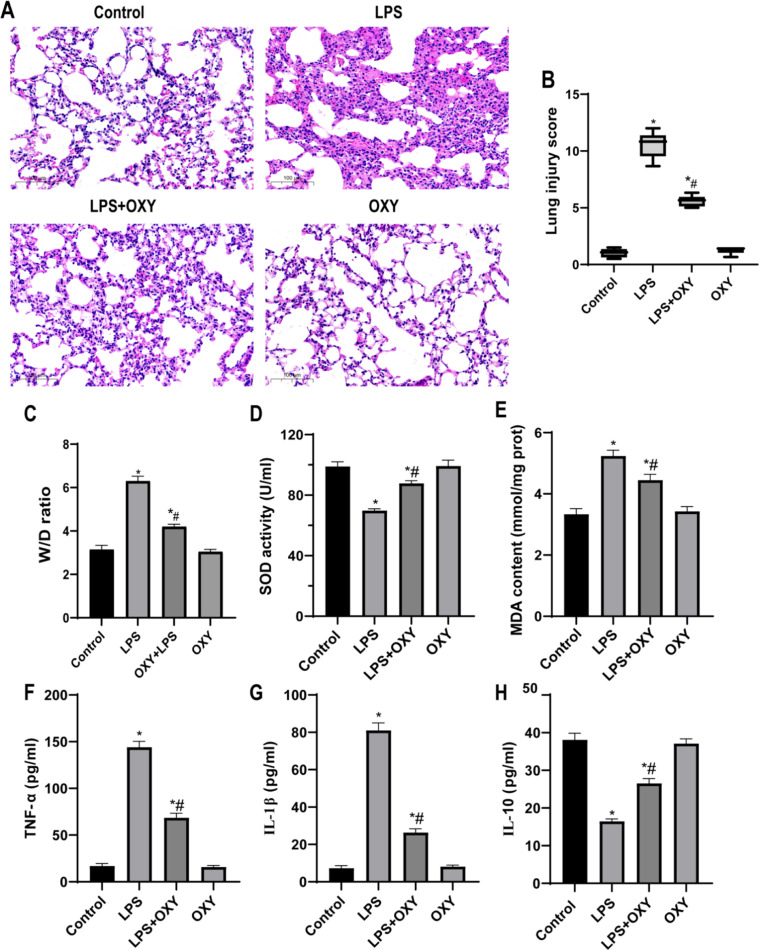
**Oxycodone alleviated LPS-driven lung pathologiccal injury and oxidative stress in mice.** (A) The histopathologic changes of lung sections stained with HE in control group, OXY group, LPS group and OXY + LPS group (original magnification, ×200), scale bar: 100 μm. (B) Lung injury scores evaluated by two blinded pathologists. Values are expressed by medians (range) using the Mann-Whitney *U* test. (C) The lung Wet/Dry (W/D) weight ratio. (D‒E) Quantitative determination of MDA and SOD concentrations. (F‒H) The serum levels of inflammatory cytokines TNF-α and IL-1β, and anti-inflammatory cytokine IL-10 levels were detected by ELISA. Date in C-H bar graph were represented as mean ± SEM using one-way ANOVA and Bonferroni test (n = 6). * p < 0.05, vs. Control group; # p < 0.05, vs. LPS group. Abbreviations: LPS, Lipopolysaccharide; HE, Hematoxylin and Eosin; ALI, Acute Lung Injury; MDA, Malondial-Dehyde; SOD, Superoxide Dismutase; TNF-α, Tumor Necrosis Factor-α; IL-1β, Interleukin-1β; IL-10, Interleukin-10; SEM, Standard Error of the Mean; OXY, Oxycodone; ANOVA, Analysis of Variance; ELISA, Enzyme-Linked Immunosorbent Assay.

In the LPS group, some obvious pathological injury was observed, such as pulmonary edema, alveolar congestion, neutrophil infiltration or aggregation, thickened alveolar wall and formation of hyaline membrane. These pathological changes were significantly improved in the oxycodone pre-treated group (Fig. 1A). In addition, the lung injury scores of the LPS + OXY group were significantly lower than those of the LPS group, as shown in Fig. 1B. The W/D ratio of the lung was used to assess the degree of pulmonary edema, and the results showed that the W/D ratio was significantly higher in LPS group than that in control group, but it was significantly lower after pre-treatment with OXY (Fig. 1C). Moreover, the MDA levels were increased while the activity of SOD was decreased in LPS group compared with the Control group, but the pre-application of oxycodone can significantly reverse the above phenomenon (Fig. 1D‒E). The levels of TNF-α, IL-1β, and IL-10 in the lung tissue are shown in Figs. 1F‒H. The results indicated that the levels of TNF-α and IL-1β were increased, while those of IL-10 were decreased in the LPS group compared with the Control group. Oxycodone administration resulted in a significant variation of these inflammation indices, which was concretely expressed as a lower level of TNF-α and IL-1β, and a higher level of IL-10 in the LPS + OXY group than in the LPS group. All of the above indicators did not change between the OXY group and the control group.

### Oxycodone reduced cell apoptosis and mitochondrial injury in lung of mice

**Fig. 2:**
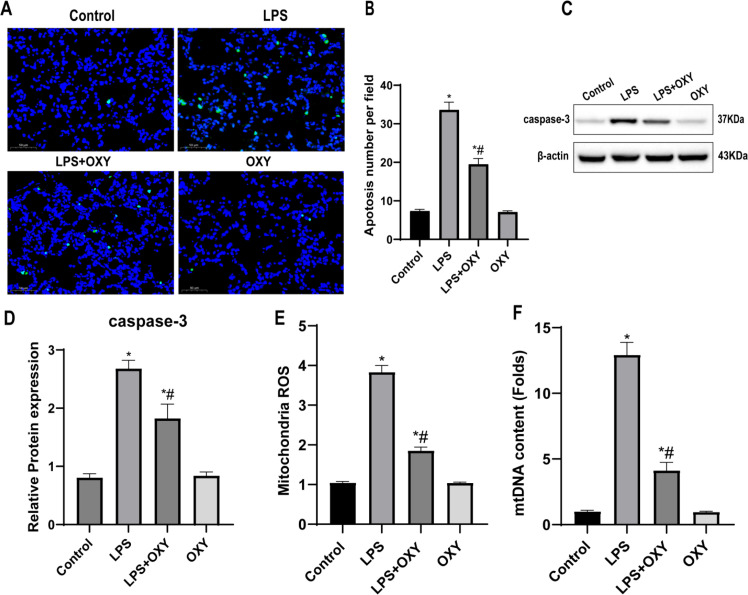
**Oxycodone reduces cell apoptosis and mitochondrial injury in mice.** (A) TUNEL staining for cell apoptosis in lung tissues. (B) Apoptosis cell number per field. The number of TUNEL-positive cells was observed by a blinded pathologist (original magnification, ×400). Scale bar: 50 μm. (C‒D) Representative western blot band and quantification to assess the expression of caspase-3 in the lung tissues (n = 3). Β-actin served as a standard for protein loading. (E) Mitochondrial ROS production was detected spectro-fluorometrically using DCFH-DA as a fluorescent dye. (F) The mtDNA content determined by real-time PCR. Date are presented as mean ± SEM using one-way ANOVA and Bonferroni test (n = 6). * p < 0.05, vs. Control group; # p < 0.05, vs. LPS group. Abbreviations: ANOVA, Analysis of Variance; TUNEL, TdT-mediated dUTP Nick-End Labeling; ROS, Reactive Oxygen Species; DCFH-DA, 2’,7’-Dichlorofluorescein Diacetate; mtDNA, Mitochondrial DNA; SEM, Standard Error of the Mean.

The authors evaluated pulmonary cell apoptosis by TUNEL assay. As shown in Fig. 2A, swelling and lytic cell death were increased in the LPS group, but the number of cell deaths was reduced in the LPS + OXY group. The percentage of apoptotic cells in the LPS + OXY group was significantly decreased compared with the LPS group (Fig. 2B). Caspase-3 was chosen as the representative protein of apoptosis and the protein levels were measured by western blot. Compared with the Control group, LPS significantly up-regulated the protein expression of caspase-3, while oxycodone can down-regulated the protein expression of caspase-3 compared with the LPS group (Fig. 2C‒D). The excessive ROS production and increased mtDNA contents was a sign of mitochondrial dysfunction. The present results showed that they were higher in the LPS group, but both of them could be significantly inhibited by oxycodone (Fig. 2E‒F). As expected, these results did not differ between OXY group and Control group.

### Oxycodone affected the expression of mitophagy-related proteins and increased the expression of HO-1 in mice

**Fig. 3:**
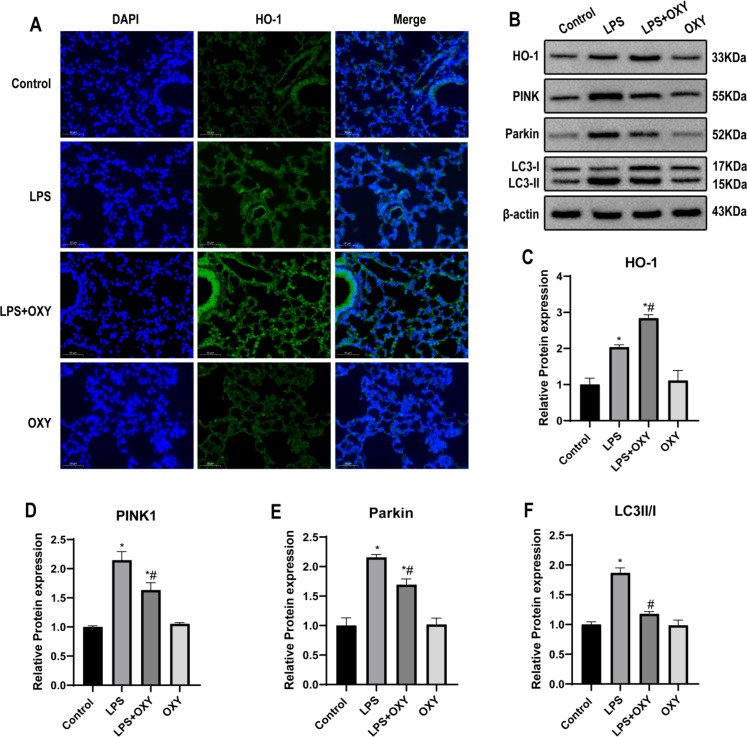
**Oxycodone pre-treatment mitigated mitophagy and activated the expression of HO-1 in LPS-stimulated lung tissue of mice.** (A) Immune-fluorescence staining of HO-1 (green) and DAPI (blue) in the four groups by fluorescence microscopy (original magnification, ×400). Scale bar: 50 μm. (B‒F) Representative western blot bands and quantification to assess the expression of mitophagy-related proteins (PINK1, Parkin, and LC3) and HO-1 in the lung tissues (n = 3). Β-actin served as a standard for protein loading. Values are expressed as mean ± SEM using one-way ANOVA corrected with Bonferroni test for multiple comparisons. * p < 0.05, vs. Control group; # p < 0.05, vs. LPS group. Abbreviations: ANOVA, Analysis of Variance; DAPI, 6-Diamino-2-Phenylindole; LC3, Microtuble-associated protein Light Chain-3; HO-1, Heme Oxygenase-1; PINK1, PTEN Induced Putative Kinase-1; SEM, Standard Error of the Mean.

The authors used the HO-1 antibody (FITC-labeling, green) and DAPI (nuclear staining, blue) to detect the expression of HO-1. The fluorescence intensity of HO-1in the LPS group was stronger than that in the Control group. Furthermore, pre-treatment with oxycodone can further increase the fluorescence intensity of HO-1 (Fig. 3A). The authors chose PINK1, Parkin, and LC3 as the representative proteins of mitophagy and measured those protein levels by western blot. Compared with the Control group, LPS significantly upregulated the protein expression of PINK1, Parkin, and LC3Ⅱ/Ⅰ. However, Pre-treatment with oxycodone can down-regulate the protein expression of PINK1, Parkin, and LC3II/I compared with the LPS group. Interestingly, the protein expression of HO-1 was significantly up-regulated after LPS challenge, and it was more obvious in the group pre-treatment with oxycodone than in the LPS group (Fig. 3B‒F).

### HO-1 deficiency impaired the protective effect of oxycodone on lung injury in mice

**Fig. 4:**
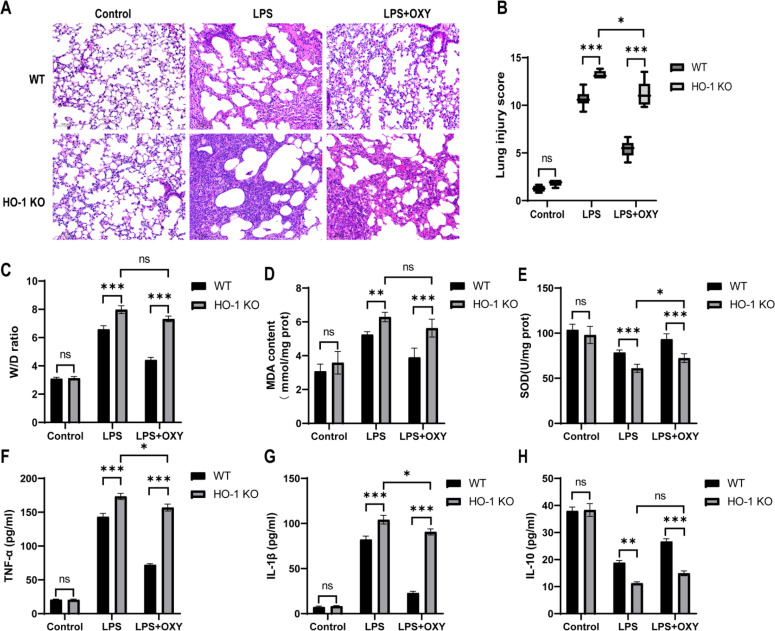
**Knockout of HO-1 weaken the protective effect of oxycodone on LPS-induced ALI *in vivo*.** (A) The representative images of histopathologic changes in lung sections with HE staining (original magnification, ×200), scale bar: 100 μm. (B) Lung injury scores determined by two blinded pathologists. (C) The lung Wet/Dry (W/D) weight ratio. (D‒E) Quantitative determination of MDA and SOD concentrations of the six groups. (F‒H) Serum levels of inflammatory cytokines TNF-α and IL-1β and anti-inflammatory cytokine IL-10 detected by ELISA. The date in B are expressed by medians (range) using the Mann-Whitney *U* test. Date C-H are presented as mean ± SEM using two-way ANOVA and Bonferroni test (n = 6). Ns, No statistical difference, * p < 0.05, ** p < 0.01, *** p < 0.001. TNF-α, Tumor Necrosis Factor-α; IL-1β, Interleukin-1β; IL-10, Interleukin-10.

HO-1 KO mice were used to investigate the role of HO-1 in oxycodone-mediated protection against ALI. The authors found that the lung injury was aggravated in HO-1 KO mice compared with the Wild-Type (WT) mice. Specifically, the knockout of HO-1 led to more severe lung histopathological injury, increased oxidative stress and inflammatory response in the HO-1 KO + LPS group than that in the WT + LPS group (Fig. 4A‒H). These results indicate that HO-1 plays a protective role against endotoxin-induced ALI. The authors further compared these indicators between WT + LPS + OXY group and HO-1 KO + LPS + OXY group. The pathological injury such as pulmonary edema, neutrophil infiltration or aggregation, and thickened alveolar wall were more severe in HO-1 KO + LPS + OXY group than that in WT + LPS + OXY group, which was consistent with the lung injury scores (Fig. 4A‒B). The W/D ratio was significantly higher in HO-1 KO + LPS + OXY group than that in WT + LPS + OXY group (Fig. 4C). Two-way ANOVA analysis revealed that there was a significant interaction effect between WT and HO-1 KO group. Specifically, the MDA levels are higher while the activity of SOD was lower in HO-1 KO + LPS + OXY group than that in WT + LPS + OXY group (Fig. 4D‒E). Moreover, higher levels of TNF-α and IL-1β and lower levels of IL-10 were observed in HO-1 + LPS + OXY group than that in WT + LPS + OXY group (Fig. 4F‒H). The authors also observed that the pre-application of oxycodone in HO-1 KO + LPS + OXY group significantly reduced the lung injury scores, increased the levels of activity SOD and decreased the levels of TNF-α and IL-1β than that in HO-1 KO + LPS group, but the contents of MDA and IL-10 had no significant differences between the two groups (Fig. 4B‒H). These results validated that knockout of HO-1 diminished the protective effects of oxycodone against LPS-stimulated ALI *in vivo*.

### HO-1 deficiency weakens the effect of oxycodone on the expression of mitophagy-related proteins

**Fig. 5:**
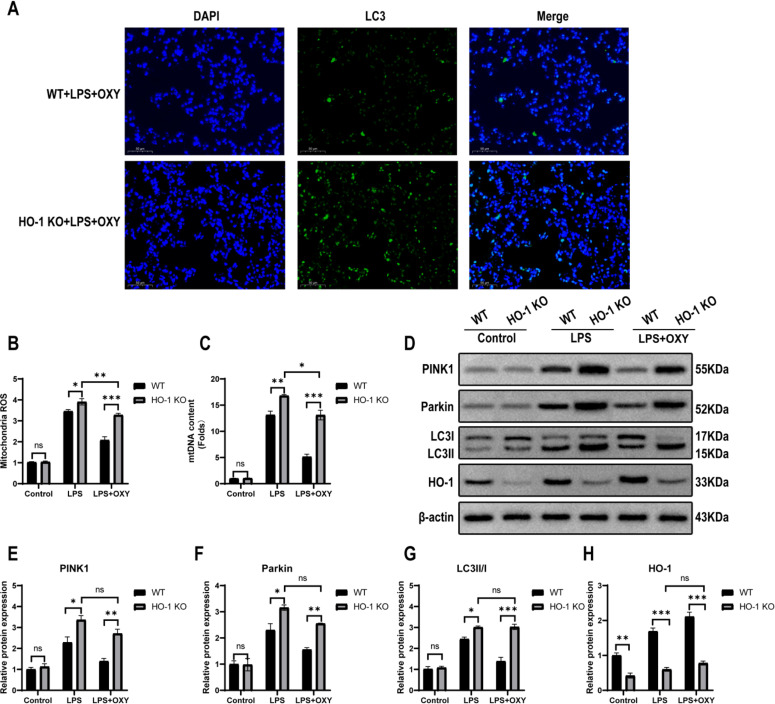
**Knockout of HO-1 inhibited the effect of oxycodone on attenuating mitophagy during ALI after LPS challenge in mice.** (A) Immunofluorescence assays of LC3 protein in lung tissue by fluorescence microscope (original magnification, ×400). Scale bar: 50 μm. LC3 and DAPI was stained with green and blue respectively. (B) Mitochondrial ROS production was detected by fluorescence spectroscopy with DCFH-DA. (C) The mtDNA content was deteceted by real time PCR. (D‒H) Representative western blot bands and quantification of mitophagy-related proteins (PINK1, Parkin, and LC3) as well as the HO-1 protein. Date are presented as mean ± SEM using two-way ANOVA and Bonferroni test for multiple comparisons (n = 3). Ns, No statistical difference, * p < 0.05, ** p < 0.01, *** p < 0.001. Abbreviations: ANOVA, Analysis of Variance; HO-1, Heme Oxygenase-1; ALI, Acute Lung Injury; ROS, Reactive Oxygen Species; DCFH-DA, 2’,7’-Dichlorofluorescein Diacetate; PINK1, PTEN Induced Putative Kinase-1; mtDNA, Mitochondrial DNA; SEM, Standard Error of the Mean; LPS, Lipopolysaccharide; OXY, Oxycodone.

To evaluate the effect of the HO-1 pathway on oxycodone pre-treatment to attenuate mitophagy, double immunofluorescence with LC3 antibody and DAPI was used. Compared with the WT + LPS + OXY group, the fluorescence intensity of FITC-LC3 was increased in the HO-1 KO + LPS + OXY group (Fig. 5A). Two-way ANOVA analysis revealed that there was a significant interaction effect between WT and HO-1 KO group. Subsequent analysis showed that ROS production and mtDNA levels were both increased after LPS challenge in WT mice and HO-1 KO mice, and knockout of HO-1 can further increase these indicators, but oxycodone can partially reverse these changes (Fig. 5B‒C). With respect to the expression of mitophagy-related proteins, PINK1, Parkin, and LC3II/I were increased in HO-1 KO + LPS group than in WT + LPS group, indicating that HO-1 KO aggravated LPS-induced mitophagy. Compared with WT + LPS + OXY mice, HO-1 KO + LPS + OXY mice showed a more remarkable increase in the protein expression of PINK1, Parkin, and LC3II/I. However, the protein expression of PINK1, Parkin, and LC3II/I had no significant differences between HO-1 KO + LPS + OXY group and HO-1 KO + LPS group (Fig. 5D‒G). These results showed that the alleviation of mitophagy by oxycodone in LPS-induced ALI was partially offset by HO-1 KO. Additionally, HO-1 expression of WT mice was increased in LPS group and was further enhanced in LPS + OXY group. However, the HO-1 expression was no statistical difference among the three groups in HO-1 KO mice (Fig. 5D and H).

### Oxycodone alleviated LPS-induced lung epithelial cell injury in mice in vitro

**Fig. 6:**
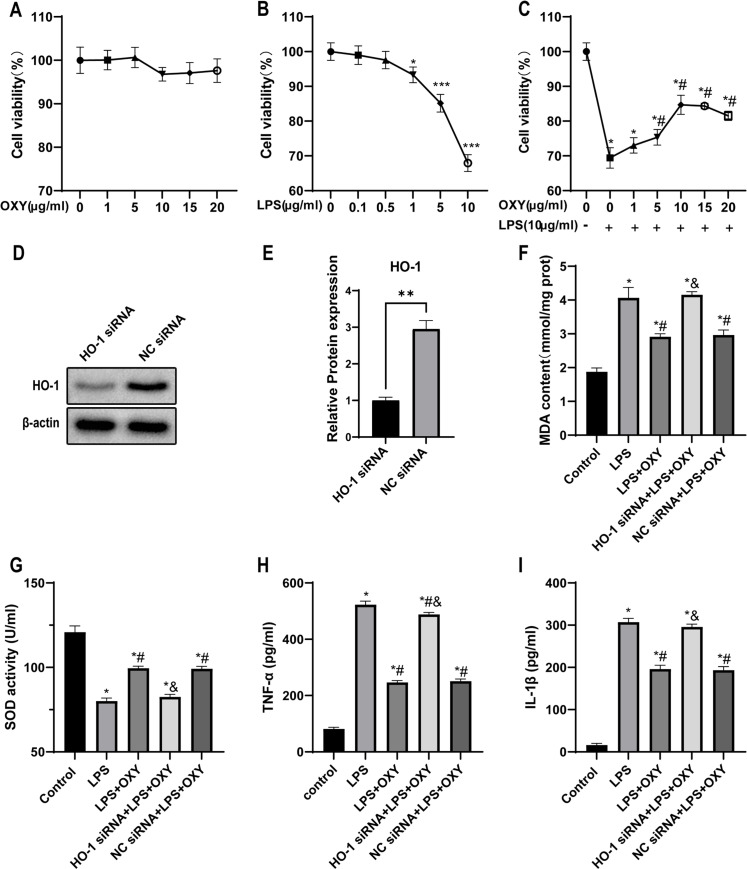
**HO-1 knockdown neutralized the effects of oxycodone in alleviating LPS-induced inflammation and oxidative injury *in vitro*.** (A) The viability of oxycodone-treated MLE12 cells was measured by MTT assays. (B) Cell viability treated with different concentrations of LPS for 24h, and was determined by MTT assays. *p < 0.05, vs. LPS = 0 group; ** p < 0.01, vs. LPS = 0 group; *** p < 0.001, vs. LPS = 0 group. (C) Viability of MLE12 cells cultivated with different concentrations of oxycodone before LPS treatment was determined by MTT assays. * p < 0.05, vs. OXY = O and LPS = 0 group; # p < 0.05, vs. OXY = O and LPS = 10 μg/mL group. (D‒E) The western blot image and quantification of HO-1 protein in MLE12 cells transfected with HO-1 siRNA and negative control siRNA (NC siRNA). ** p < 0.01. (F‒G) Levels of MDA and SOD in the five groups. (H‒I) Levels of inflammatory factors TNF-α and IL-1β in the cell supernatant. The date of F‒I were expressed as mean ± SEM using one-way ANOVA and Bonferroni test for multiple comparisons (n = 6). * p < 0.05, vs. Control group; # p < 0.05, vs. LPS group; & p < 0.05, vs. LPS + OXY group.

To select the most appropriate oxycodone concentration, the authors first performed experiments to test cell viability after LPS challenge with different oxycodone concentrations by an MTT assay. As shown in Fig. 6A, the different concentrations of oxycodone had no effect on cell viability, indicating that oxycodone had no cytotoxicity on cells. To simulate a cellular model of LPS-induced ALI, MLE12 cells were co-cultured with different concentrations of LPS, and the cell viability in different groups was shown in Fig. 6B. After treatment with 10 μg/mL LPS for 24h, the viability of MLE12 cells was significantly lower than that of untreated cells. Thus, the LPS concentration of 10 μg/mL was used to create the cell model. The follow-up experiments showed that pre-treatment with oxycodone can significantly alleviate the LPS-induced decline in cell viability. The authors also observed that oxycodone could significantly reduce the LPS-induced decrease in cell viability at a concentration of 10 μg/mL (Fig. 6C). Therefore, the authors performed the following experiments with oxycodone at a dose of 10 μg/mL. Then, the effect of HO-1 gene silencing was evaluated by western blotting, and the results showed that the protein expression of HO-1 in the HO-1 siRNA group was significantly down-regulated compared with the NC siRNA group (Fig. 6D‒E). Compared with the Control group, the MDA levels were increased while the activity of SOD was decreased in the LPS group. However, Pre-treatment of oxycodone significantly reduced the levels of MDA and increased the SOD levels in LPS + OXY group. However, oxycodone did not have a protective effect on the HO-1 siRNA group (Fig. 6F‒G). In addition, TNF-α and IL-1β were elevated after LPS stimulation, and the levels of these inflammatory markers were significantly reduced by pre-administration of oxycodone. However, this protective effect of oxycodone was not observed in the HO-1 gene silencing group (Fig. 6H‒I), indicating that HO-1 knockdown may partially attenuate the anti-inflammatory effects of oxycodone on LPS-treated cells.

### Oxycodone attenuates LPS-induced cell injury by regulating mitophagy via the HO-1 pathway

**Fig. 7:**
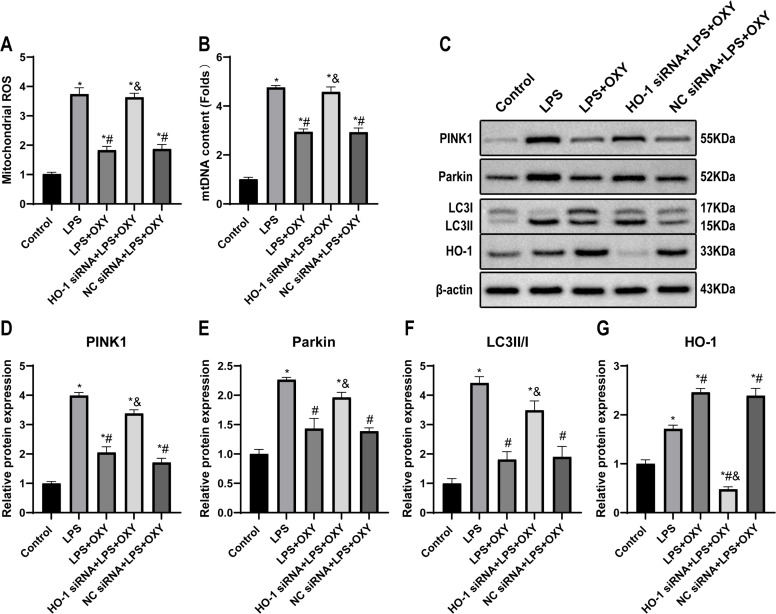
**Oxycodone inhibited LPS-induced MLE12 cells damage by regulating mitophagy through the HO-1 pathway.** (A) Mitochondrial ROS production was measured by fluorescence spectroscopy using DCFH-DA as the fluorescent dye. (B) The mtDNA content detected by real time PCR. (C‒G) The representative western blot bands and quantification of mitophagy-related proteins (PINK1, Parkin, and LC3) and pathway-related protein (HO-1). Band intensity analysis of western blotting images was performed using their relative ratios to β-actin. Date were expressed as mean ± SEM using one-way ANOVA and Bonferroni test for multiple comparisons (n = 3). * p < 0.05, vs. Control group; # p < 0.05, vs. LPS group; & p < 0.05, vs. LPS + OXY group. Abbreviations: ANOVA, Analysis of Variance; ROS, reactive oxygen species; DCFH-DA, 2’,7’-Dichlorofluorescein Diacetate; mtDNA, Mitochondrial DNA; PINK1, PTEN induced putative kinase 1; HO-1, Heme Oxygenase-1; KO, Knockout; SEM, Standard Error of the Mean; LPS, Lipopolysaccharide; OXY, Oxycodone.

As suspected, the levels of ROS and mtDNA were increased after LPS stimulation, but this phenomenon could be significantly reversed by oxycodone. However, HO-1 deficiency significantly weakens the protective effect of oxycodone (Fig. 7A‒B). The expression of mitophagy-related proteins PINK1, Parkin, and LC3 are shown in Figs. 7C‒F. Compared with the control group, the authors observed a significant increase in the expression of PINK1, Parkin and LC3Ⅱ/Ⅰ in LPS group, but they were decreased by oxycodone pre-treatment. Unsurprisingly, HO-1 knockdown blocked the oxycodone-mediated regulatory function of mitophagy. Specifically, the levels of PINK1, Parkin and LC3Ⅱ/Ⅰ were remarkably elevated in the HO-1 siRNA group than those in LPS + OXY group, which is consistent with the effect of oxycodone *in vivo*. As shown in Figs. 7C and 7G, HO-1 expression was increased in LPS group than that in the control group, and it was further up-regulated HO-1 protein expression after pre-treatment with oxycodone. Compared with the oxycodone pre-treatment group, the negative control siRNA did not affect any variables.

## Discussion

**Fig. 8:**
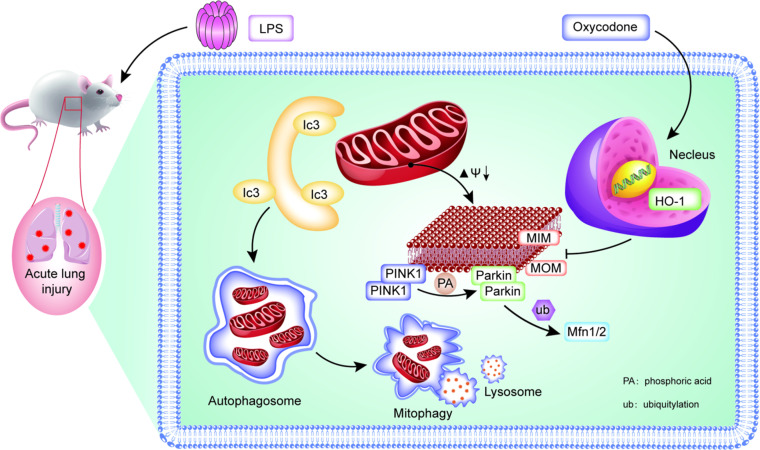
**Graphical illustration of oxycodone pretreatment-mediated suppression of mitophagy in LPS-induced ALI or cell damage by regulating the HO-1 pathway.** Oxycodone activated the HO-1 pathway when LPS was administered, leading to the response to induce target gene of HO-1 expression. Additionally, pretreatment with oxycodone downregulated the expressions of mitophagy markers PINK1, Parkin, and LC3Ⅱ/Ⅰ. In conclusion, oxycodone pre-treatment alleviates mitophagy in endotoxin-related ALI and cell damage, thus alleviating the inflammation and oxidative stress of lung tissue and cells, ultimately reducing cell death.

The present study showed that oxycodone can attenuate LPS-induced ALI by regulating mitophagy via the HO-1 pathway. Systemic LPS administration is a widely used model for studying Systemic Inflammatory Response Syndrome (SIRS) and its progression to ALI. Given that oxycodone was administered as a pretreatment, its observed protective effects may be attributed to a reduction in SIRS rather than being specific to lung injury. At present, there are still some extensive studies on the effective treatment and pathogenesis of ALI, and the earlier studies have identified HO-1 as an important protein against endotoxin-induced ALI.^17^^,^^17^^,^^27^^,^^28^ Some previous studies showed that oxycodone can inhibit inflammation and apoptosis^29^^,^^30^ but the specific underlying mechanism needs to be further explored. To our knowledge, this is the first study to report that oxycodone can alleviate endotoxin-induced ALI by attenuating inflammation and oxidative stress via down-regulating mitophagy. Moreover, the authors also find that the protective effect of oxycodone depends on the HO-1 pathway (Fig. 8). Therefore, the present findings may provide a new target for the treatment of endotoxin-induced ALI.

Sepsis, a life-threatening syndrome, is the second leading cause of mortality in intensive care units.^31^ Due to the occurrence of ALI, the lung is the first organ to fail in patients with sepsis.^32^ In the present study, the authors constructed an endotoxin-induced ALI model. These results showed that administration of LPS can induce lung injury *in vivo*, specifically manifested as pathological lung injury. With the application of LPS, the levels of oxidative stress index MDA were increased while the activity of SOD was decreased, followed by the elevated inflammatory factors such as TNF-α and IL-1β and the reduced anti-inflammatory cytokine IL-10. Moreover, the percentage of apoptotic cells in the lung tissue was elevated after LPS challenge.

Mitophagy eliminates damaged mitochondria or defective mitochondria; thus, it plays an important role in mitochondrial quality control and cellular homeostasis.^33^ However, the dysregulation of mitophagy is implicated in various human diseases.^8^ Previous studies have found that mitophagy promotes the clearance of damaged mitochondria and reduces the production of ROS in LPS-induced ALI, thereby alleviating lung injury.^34^ However, the excessive mitophagy may lead to a lack of healthy mitochondria, eventually leading to cell damage and death.^11^^,^^35^ Therefore, mitophagy has a dual effect on ALI. The oxidative stress and inflammation induced by LPS in the lung can lead to enhanced mitophagy. As these results showed that the levels of mitochondrial ROS and mtDNA were increased after LPS challenge, which could result in mitochondrial dysfunction. Mitophagy-related signaling pathway involves PINK1/Parkin, Bcl-2 homology-3 (BH3)-only protein Nix (Bnip3L),^36^^,^^37^ FUN14 Domain Containing 1 (FUNDC1), Bcl-2-Like protein 13(BCL-2L13), and several triggers like lipids and Beclin1 Regulator (AMBRA-1).^38^^,^^39^ The PINK1/Parkin mediated pathway is the canonical mechanism underlying mitophagy. PINK1 stabilizes on Mitochondrial Outer Membranes (MOM) and phosphorylates various proteins such as Parkin and ubiquitin, while Parkin ubiquitinates MOM proteins.^40^ Parkin mediates the formation of polyubiquitin chains, which are recognized by LC3 chains and form complexes. Then, it enfolds the mitochondria to form mitochondrial autophagosomes, which are eventually recognized and degraded by lysosomes.^41^ LC3 is a reliable autophagosome marker; in addition, the authors also chose PINK1 and Parkin as the representative proteins of mitophagy in the present study. Consistently, after LPS-stimulation, the expression of protein of PINK1 and Parkin was up-regulated, accompanied by the increased conversion of LC3-Ⅱ to LC3-Ⅰ, indicating that mitophagy is enhanced. Therefore, inhibition of excessive mitophagy is essential to reduce mitochondrial damage and protect mitochondrial function.

Oxycodone, as a semi-synthetic opioid, has been widely used to treat post-operative and other acute pain in adults, as it can modulate cytokine levels, inhibit inflammation and oxidative stress.^42^ In addition to the research on pain treatment, the current research on oxycodone also includes the protection of the heart, brain and lung.^29^^,^^43^ Based on previous studies^23^^,^^44^ and the pre-experimental results, the authors set the dosage of oxycodone at 2 mg/kg. Accordingly, the present results showed that pre-treatment with oxycodone before LPS challenge can alleviate the LPS-driven pulmonary damage, which was supported by lower lung injury scores and W/D ratio, reduced cell apoptosis, decreased contents of MDA, and increased activity of SOD, and an overall lower level of inflammatory factors. In addition, the authors found that the pre-administration of oxycodone down-regulated the expression of mitophagy-related proteins such as PINK1, Parkin, and LC3Ⅱ/Ⅰ, accompanied by decreased content of mitochondrial ROS and mtDNA. These results suggested that oxycodone can alleviate the LPS-induced lung injury and cell damage, and the mechanism may be related to the suppression of mitophagy.

HO-1 is a common stress-inducing protein of mammals that prevents oxidative cell injury. It is induced by some specific factors, such as cytokines, endotoxins, and ROS, to produce endogenous carbon monoxide, which plays an anti-inflammatory, anti-apoptotic, and cytoprotective role.^37^^,^^45^ In the previous studies of the research group, the authors found that HO-1 can alleviate endotoxin-induced ALI by maintaining the homeostasis of mitochondrial fusion/fission and inhibiting pyroptosis and Golgi stress.^7^^,^^17^^,^^27^ In the present study, the authors found that oxycodone pre-application could increase the expression of HO-1 in lung tissue. Thus, the authors speculated whether the protective effect of oxycodone against LPS-induced lung injury might be via the HO-1 pathway. Therefore, the authors performed the follow-up experiments to test this hypothesis by using HO-1 knockout mice and HO-1 siRNA in MLE12 cells. The present results showed that with HO-1 knockout, the LPS-induced lung injury was more severe than that in WT mice, as evidenced by higher lung injury scores, increased contents of MDA, and decreased activity of SOD, indicating that HO-1 may play a protective role in lung injury. The pre-treatment with oxycodone significantly alleviated LPS-induced lung injury in WT mice, but no similar effect was observed in HO-1 knockout mice. In addition, the authors observed that pretreatment with oxycodone significantly suppressed the functional impairment of mitochondria as well as mitophagy. Specifically, ROS and mtDNA were decreased, while PINK1, Parkin, and LC3Ⅱ/Ⅰ protein levels were lower. However, these protective effects were not observed in the HO-1 KO model, suggesting that oxycodone exerts a protective effect against LPS-induced lung injury by regulating mitophagy, and it needs to be realized via the HO-1 pathway. Consistent with the above indicators in the lung, the application of oxycodone could reverse the cell damage induced by LPS, manifested by increased cell viability, lower contents of MDA, and higher levels of SOD followed by decreased inflammatory factors TNF-α and IL-1β. Besides, mitochondrial ROS production and mtDNA contents were also decreased. In addition, pretreatment with oxycodone enhanced the expression of HO-1 and simultaneously down-regulated the expression of PINK1, Parkin, and LC3Ⅱ/Ⅰ, indicating that oxycodone alleviated LPS-induced cell damage by reducing the occurrence of mitophagy. However, with the loss of HO-1 by siRNA in LPS-exposed MLE12 cells, the above-mentioned maintenance effects of oxycodone were significantly inhibited, which also proved that HO-1 is the key pathway via which oxycodone reduces mitophagy levels to improve LPS-induced cell damage.

In conclusion, oxycodone could effectively attenuate the endotoxin-induced ALI and cell damage by regulating mitophagy via the HO-1 pathway.

However, the present study has some limitations. Firstly, while systemic LPS administration is a well-established model for studying SIRS and its progression to ALI, the effects observed in this study, given that oxycodone was used as a pretreatment ‒ may primarily reflect a reduction in systemic inflammation rather than lung-specific mechanisms. Future studies using direct lung injury models (e.g., intratracheal LPS) could help isolate the lung-specific effects of oxycodone. Although the model reflects systemic inflammation, the protective effects of oxycodone on lung injury suggest its potential utility in clinical settings where systemic inflammation contributes to ALI, such as sepsis. Further studies are needed to confirm these effects in lung-specific injury models. Secondly, the authors only verified the association between oxycodone and the HO-1 pathway, and we did not further explore its upstream or downstream pathway. In future studies, the authors will continue to explore the precise molecular mechanisms of HO-1 in oxycodone-regulated mitophagy in ALI. Thirdly, the authors only silenced the HO-1 gene to explore the underlying mechanism of oxycodone in endotoxin-induced ALI without activating the HO-1 pathway. Fourthly, in order to observe the effect of oxycodone on normal lungs and reduce the mortality in mice, the authors chose to pretreat the mice with oxycodone before the administration of LPS. In the follow-up experiment, the authors will try to use oxycodone after the occurrence of ALI to see its effect. Additionally, whether oxycodone is involved in other processes such as pyroptosis and endoplasmic reticulum stress still needs to be further studied.

## Conclusion

The authors demonstrated that oxycodone could attenuate LPS-stimulated ALI by down-regulating mitophagy via the HO-1 signaling pathway, both *in vivo* and *in vitro*. Selective suppression of the HO-1 pathway can reverse the effect of oxycodone. These findings might provide a potential therapeutic option for sepsis patients with ALI.

## Ethics statement

All animal care and experimental procedures of mice were conducted according to the National Institutes of Health Guidelines with the approval of the Animal Ethical and Welfare Committee of Tianjin Nankai Hospital (Tianjin, China, approved number: NKYY-DWLL-2022-049).

## Funding

This research was funded by Tianjin key Medical Discipline (Anesthesiology) Construction Project and the 10.13039/501100006606Natural Science Foundation of Tianjin, number 21JCYBJC01240.

## CRediT authorship contribution statement

**Cuicui Liu:** Data curation, Formal analysis, Writing – original draft. **Yanting Wang:** Software, Methodology, Writing – original draft. **Shaona Li:** Project administration, Writing – review & editing. **Jia Shi:** Funding acquisition, Writing – review & editing. **Pei Wang:** Software, Validation, Writing – review & editing. **Yang Ma:** Methodology, Project administration, Writing – review & editing. **Xiangkun Li:** Data curation, Formal analysis, Project administration, Writing – review & editing. **Jianbo Yu:** Conceptualization, Writing – review & editing.

## Declaration of competing interest

The authors declare that the research was conducted in the absence of any commercial or financial relationships that could be construed as a potential conflict of interest.
